# Treatment of Chronic Migraine with OnabotulinumtoxinA: Mode of Action, Efficacy and Safety

**DOI:** 10.3390/toxins7072659

**Published:** 2015-07-17

**Authors:** Délia Szok, Anett Csáti, László Vécsei, János Tajti

**Affiliations:** 1 Department of Neurology, University of Szeged, Semmelweis str. 6, Szeged H-6725, Hungary; E-Mails: szok.delia@med.u-szeged.hu (D.S.); csati.anett@gmail.com (A.C.); 2 MTA—SZTE Neuroscience Research Group, Szeged H-6725, Hungary; E-Mail: vecsei.laszlo@med.u-szeged.hu

**Keywords:** chronic migraine, efficacy, mode of action, onabotulinumtoxinA, pathomechanism, safety, tolerability, treatment

## Abstract

Background: Chronic migraine is a common, highly disabling, underdiagnosed and undertreated entity of migraine. It affects 0.9%–2.2% of the general adult population. The present paper overviews the preclinical and clinical data regarding the therapeutic effect of onabotulinumtoxinA in chronic migraineurs. Methods: A literature search was conducted in the database of PubMed up to 20 May 2015 for articles related to the pathomechanism of chronic migraine, the mode of action, and the efficacy, safety and tolerability of onabotulinumtoxinA for the preventive treatment of chronic migraine. Results: The pathomechanism of chronic migraine has not been fully elucidated. The mode of action of onabotulinumtoxinA in the treatment of chronic migraine is suggested to be related to the inhibition of the release of calcitonin gene-related peptide and substance P in the trigeminovascular system. Randomized clinical trials demonstrated that long-term onabotulinumtoxinA fixed-site and fixed-dose (155–195 U) intramuscular injection therapy was effective and well tolerated for the prophylactic treatment of chronic migraine. Conclusions: Chronic migraine is a highly devastating entity of migraine. Its exact pathomechanism is unrevealed. Two-third of chronic migraineurs do not receive proper preventive medication. Recent clinical studies revealed that onabotulinumtoxinA was an efficacious and safe treatment for chronic migraine.

## 1. Introduction

Migraine is a highly disabling primary headache disorder that affects 6%–10% and 17%–25% of the adult population in men and women, respectively, with a male to female ratio of 1:3 [1,2,3]. The disease is ranked among the top 10 causes of disability worldwide [2,4]. Migraine headache is characterized by unilateral, throbbing and pulsating, moderate or severe pain, accompanied by nausea and vomiting, and photo- and phonophobia [5]. The two main subtypes are migraine without and with aura [5]. One of the most serious conditions related to migraine headache is chronic migraine (CM), which definitely influence the quality of life of the patients and represents a worldwide societal burden [6,7]. CM occurs more frequently in women than in men (2.5–6.5 times more often in female compared to male patients) [8]. The importance of CM as a disease is reflected by the fact that the International Classification of Headache Disorders (ICHD) 3 beta has recently categorized it as an independent disease entity [5]. CM patients lose 4.6 h per week because of the headache [9]. The CM-related healthcare resource was higher than that related to episodic migraine [6]. The estimated prevalence of CM ranges from 0.9% to 2.2% in the general population [10,11,12]. The definition and the diagnostic criteria of CM are the following: headache (tension-type-like and/or migraine-like) occurring on 15 or more days per month for more than three months, fulfilling the criteria of migraine with or without aura on at least eight days per month [5]. The exact pathomechanism of CM is still unrevealed; however, altered brain energy metabolism and central sensitization process have been postulated to play crucial roles in its development [13]. The treatment of CM is likewise unsolved. Only around 33% of migraine patients receive proper preventive migraine treatment, e.g., antiepileptic drugs (topiramate, valproic acid/sodium valproate) or antidepressants (amitriptyline) [10,14,15,16]. In October 2010, the Food and Drug Administration (FDA) approved onabotulinumtoxinA (OBOT-A) fixed-site and fixed-dose (155–195 U) intramuscular injection as a preventive treatment for CM [17]. The supposed mode of action of OBOT-A in CM includes the inhibition of calcitonin gene-related peptide (CGRP) and substance P (SP) release in the trigeminovascular system (TS) [8,13]. The pooled analysis of Phase III REsearch Evaluating Migraine Prophylaxis Therapy (PREEMPT)-1 and PREEMPT-2 studies revealed that long-term treatment with OBOT-A was highly effective in reducing headache frequency and was generally safe and well tolerated as a prophylactic medication of CM [7,18,19,20].

The present paper reviews the mode of action, efficacy, safety and tolerability of OBOT-A in the prophylactic treatment of CM patients.

## 2. Materials and Methods

A literature search was conducted in the database of PubMed up to 25 May 2015 for articles related to pathomechanism of chronic migraine, the mode of action of OBOT-A and the efficacy, safety and tolerability of OBOT-A for preventive treatment of chronic migraine.

## 3. Results

### 3.1. Pathomechanism of CM

The pathomechanism of migraine is still not fully understood. One of the leading hypotheses is related to the activation of the TS [21]. The “skeleton” of the TS consists of the trigeminal ganglion (TRIG), which forms a bridge between the cortical and meningeal vasculature, and the second-order nociceptive neurons in the brainstem [21]. This hypothesis unifies the vascular and neuronal theories of migraine, defining the disease as a neurovascular disorder [22,23]. The neurobiological background of the clinical picture of migraine is proposed to include the peripheral and central sensitization within the TS. During this process, the vasoactive neuropeptides such as CGRP and pituitary adenylate cyclase-activating polypeptide (PACAP) are released from the TRIG both from the peripheral and central arches of the pseudounipolar neurons [24]. These vasoactive neuropeptides evoke neurogenic inflammation, leading to peripheral sensitization in the dura mater and central sensitization in the trigemino-cervical complex (TCC) [25,26,27,28,29]. The TCC represents a part of the brainstem, which involves the caudal part of the trigeminal nucleus caudalis (TNC) and the dorsal horn of the C1-2 segments of the cervical spinal cord [26]. Peripheral sensitization explains the throbbing nature of migraine headache and the worsening of the headache due to physical activity during a migraine attack [30,31]. On the other hand, central sensitization reflects the allodynia and the pericranial muscle tenderness during the painful headache periods [30,32,33,34].

Allodynia as a clinical marker of the central sensitization—which is an abnormal sensory state where normally innocuous stimuli are felt as painful—is a frequent accompanying sign during migraine attack [30,35,36]. The incidence of cutaneous allodynia is higher in patients with CM compared to episodic migraineurs [37]. Cutaneous allodynia might be an independent predictor of the chronification of migraine [38].

The risk of developing CM was higher in those migraine patients who had high migraine attack frequency (5–9 days *vs.* less than four days of headache per month) [39]. The other modifiable risk factors for CM include obesity (body mass index above 30 kg/m^2^), anxiety and depression, stressful life events, heavy caffeine consumption, smoking and medication overuse [40]. It has been revealed that the presence of depression (from moderate to severe intensity) is associated with an increased risk of the development of CM [41]. CM is associated with comorbid conditions, e.g., psychiatric (anxiety—30.2%, depression—30.2%), pain (chronic pain—31.5%, arthritis—33.6%), respiratory (asthma—24.4%, sinusitis—45.2%), cardiovascular (hypertension—33.7%) and metabolic (obesity—25.5%) disorders [40]. It is also a meaningful finding that the severity of allodynia was the highest in CM patients with comorbid depression [37,42]. It seems that there is a bidirectional connection between CM and episodic migraine subtypes. The AMPP (American Migraine Prevalence and Prevention) study revealed that migraine chronification occurred in association with an increased intake of abortive migraine medications [40]. *Vice versa*, the withdrawal of these drugs reversed CM state to episodic migraine [40].

The exact pathomechanism of CM is unknown [40]. There are some preclinical and clinical data pointing out the impact of altered brain structures and metabolism, cortical hyperexcitability and the central sensitization of the TS in the pathogenesis of CM. Neuroimaging studies revealed the reduction of cerebral gray matter in the pain matrix, such as that of the anterior cingulate cortex, the reduction of which showed a positive correlation with migraine attack frequency [13]. In the early 1900s, Weiller and his group elegantly demonstrated that during a spontaneous migraine attack, specific-brainstem nuclei (e.g., periaqueductal gray matter (PAG), locus coeruleus and raphe nuclei) showed increased activity, as revealed by positron emission tomography (PET) [43]. Furthermore, iron accumulation in the PAG was observed in migraine, which showed a correlation with disease duration [44]. Human PET studies concerning brain metabolism and hyperexcitability suggested that the orbitofrontal and temporal cortices might play a role in the initiation of the CM [13].

### 3.2. Supposed Mode of Action of OBOT-A in Chronic Migraine Therapy

**Figure 1:**
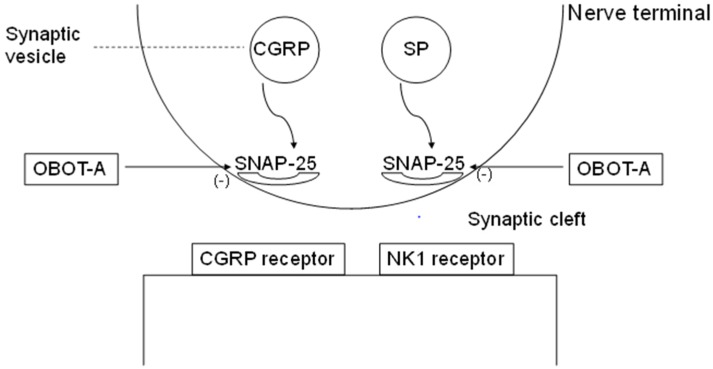
Schematic presentation of the mode of action of OBOT-A in CM. (modified by Ref. [20]) Abbreviations: CGRP, calcitonin gene-related peptide; OBOT-A, onabotulinumtoxinA; NK1, neurokinin1; and SNAP-25, synaptosomal-associated protein of 25 kDa.

Botulinum neurotoxin-A—a potent purified neurotoxin complex produced by anaerobic bacteria *Clostridium botulinum*—affects the nervous system within the neuromuscular junctions by means of a specific cleavage of the soluble *N*-ethylmaleimide-sensitive factor (NSF)-attachment protein receptor complex (SNARE)-like synaptosomal-associated protein of 25 kDa (SNAP-25) [45,46] (Figure 1). The result of this multistage process is the disruption of pain neurotransmission, including the inhibition of the release of CGRP, SP and glutamate [45].

Within the sympathetic sudomotor C nerve fiber terminals surrounding the sweat glands, acethylcholine is co-localized with CGRP [47,48]. CGRP is capable of enhancing cholinergic sweating [49,50]. Clinical examinations demonstrated that botulinum toxin treatment can reduce focal (palmar and axillary) hyperhidrosis with a relatively long duration of effect [51]. CGRP is widely distributed in the sensory system, including also the dorsal root ganglia and the trigeminal ganglia [52,53,54]. Preclinical and clinical studies revealed that botulinum neurotoxin exerted analgesic effect in inflammatory as well as neuropathic pain via direct and indirect peripheral and central nociceptive pathways through the inhibition of the release of neuropeptides such as CGRP [55].

**Figure 2:**
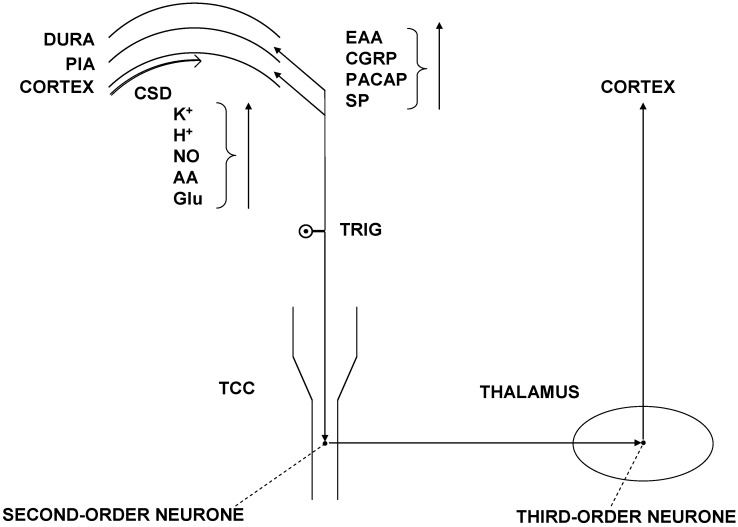
Schematic presentation of the trigeminovascular system. (modified by Refs. [22,23,26]) Abbrevations: CGRP, calcitonin gene-related peptide; CSD, cortical spreading depression; LC, locus coeruleus; NRM, nucleus raphe magnus; PACAP, pituitary adenylate-cyclase activating polypeptide; PAG, periaqueductal gray matter; SP, substance P; TCC, trigemino-cervical complex; and TRIG, trigeminal ganglion.

Preclinical studies revealed that OBOT-A has direct effect on peripheral sensitization and indirect effect on central sensitization [13]. OBOT-A inhibits evoked (by potassium chloride or capsaicin), but not basal (unstimulated) release of CGRP in rat TRIG cell culture model [56]. Moreover, the proton-regulated release of CGRP from cultured primary trigeminal ganglion neurons utilizes a different mechanism than the calcium- and SNAP-25-dependent pathways, which mechanism in this case cannot be inhibited by OBOT-A [57]. Botulinum toxin influences the release of SP in embryonic rat dorsal root ganglion neurons [58]. Recently, it has been revealed that OBOT-A inhibits mechanical nociception in peripheral trigeminovascular neurons [59]. CGRP has a crucial role in the peripheral and central sensitization in migraine [24,25,60,61]. Cortical spreading depression (CSD) is a strong, slowly-propagating depolarization of neuronal and glial elements of the cortex, accompanied by the depression of electroencepehalographic activity and by a high amount of increase in the levels of extracellular potassium ion [26]. In the peripheral part of the TS (*i.e.*, vasculature of the cerebral dura mater and pia mater), the consequences of the CSD include the stimulation of trigeminal nociceptors. After this process, vasoactive neuropeptides such as CGRP, PACAP and SP are released through the peripheral branches of TRIG, leading to neurogenic inflammation and peripheral sensitization (Figure 2).

Neuron-glia interactions in the TRIG represent a novel aspect of the trigeminovascular hypothesis [62,63]. The release of CGRP during neuronal activation of the TRIG stimulates the satellite glia cells, which in turn release proinflammatory cytokines, thereby further modulating the neuronal response [62,63,64,65]. This process is suggested to participate in the maintenance of CM [66]. The central part of the TS projects toward the TCC and activates the second-order nociceptive neurons, which convey the information from the brainstem to the thalamus [24,26]. There are data reflecting that CGRP is involved in the activation of second-order nociceptive neurons in the TCC [24,25,60]. CGRP is able to facilitate glutamatergic neurotransmission in the dorsal horn of the spinal cord [67]. Furthermore, CGRP influences the discharge frequency of wide dynamic range neurons in the spinal cord, leading to the consequent initiation of central sensitization [60,68]. CGRP might modulate the activation of TS through its action within the PAG, where CGRP acts on calcitonin receptor-like receptor (CLR) and receptor activity-modifying protein (RAMP)-1 receptor [60].

In a recent clinical study, it has been revealed that interictal levels of CGRP in the plasma of patients with CM were decreased following OBOT-A treatment [69]. Furthermore, the authors concluded that CM patients who responded well to OBOT-A treatment had higher pretreatment CGRP plasma levels compared to non-responders [69].

CGRP is a migraine-related neuropeptide, which play roles in both the peripheral and central sensitization in the TS, as well as in the neuron-glia interaction in the TRIG. OBOT-A may inhibit the release of CGRP.

### 3.3. Therapeutic Indications of OBOT-A in Primary Headaches

The aim of migraine treatment is not just relieving pain and reducing migraine attack frequency but also preventing the progression towards the chronification of migraine [70]. The first implication suggests that the therapeutic potential of OBOT-A administration in headache treatment stems from the case of headache sufferers who reported improvement of their headaches after its cosmetic use for facial wrinkles [8,18]. After this observation, numerous (open label and randomized) clinical trials were conducted in different types of headaches (e.g., chronic daily headache, tension-type headache, episodic and CM) [18]. Finally OBOT-A has been approved only in the treatment of CM [19].

### 3.4. Technique of Injection of OBOT-A in CM

The principle of the technique using OBOT-A is the administration of fixed-site and fixed-dose injections. The required technique includes at least 31 injection sites across the head and neck regions [71]. The recommended fixed-sites and fixed-doses (totally 155–195 units (U)/cycle) are the follows: corrugator muscle: 5 U (each side), procerus muscle: 5 U (one side), frontal muscle: 10 U (each side), temporalis muscle: 20 U (each side), occipitalis muscle: 15 U (each side), cervical paraspinal muscle: 10 U (each side), trapezius muscle: 15 U (each side) [8]. The value of U represents an estimated dose of intraperitoneally injected toxin lethal for 50% of the female Swiss Webster mice (weighing 18–20 grams) [46]. The purpose of this procedure is to reach the supraorbital, supratrochlear and auriculotemporal branches of the trigeminal nerves and the greater and lesser occipital nerve branches, as well as the sensory rami from the cervical spinal segments C3-C5 [8]. The recommended needle size is a 30-gauge, 0.5-inch needle, which should be inserted at a 45-degree angle to the plane of the head and neck [8]. The repeated injection schedule is every 12 weeks [19].

### 3.5. Efficacy of OBOT-A in Treatment of CM

The largest randomized clinical trials that evaluated the efficacy of OBOT-A in CM were the PREEMPT-1 and PREEMPT-2 studies [19]. Analysis of the primary endpoint for the efficacy from the pooled data set revealed that OBOT-A significantly reduced the number of headache days per 28-day cycle relative to placebo at week 24 (−8.4 *vs.* −6.6; *p* < 0.001) [19]. The secondary endpoints were the following: mean change from baseline in frequency of migraine days (*p* < 0.001); frequency of moderate or severe headache days (*p* < 0.001); cumulative hours of headache and headache days (*p* < 0.001); frequency of headache episodes (*p* < 0.009); frequency of migraine episodes (*p* = 0.004); and the proportion of patients with a severe Headache Impact Test-6 score (*p* < 0.001) [19]. In the OBOT-A-treated group, the intake of triptans as acute pain medication was significantly less compared to the placebo group at week 24 (*p* < 0.001) [19]. In PREEMPT 24-week pooled subgroup analysis of CM patients with medication overuse revealed that the change from baseline in the frequency of acute headache medication intakes was not statistically significant, except for triptan intakes [72].

A single-center, double-blind, comparative pilot study revealed that OBOT-A and topiramate (an antiepileptic drug used for migraine prevention with Level A evidence) demonstrated similar efficacy in the prophylaxis of CM [73,74,75]. Likewise, a multi-center, double-blind, comparative pilot study demonstrated that OBOT-A and topiramate had similar efficacy (by week 26 in the reduction of headache days per month compared with baseline) in patients with CM [76]. The secondary subgroup analysis of the data of CM patients who received all five treatment cycles of OBOT-A and completed the PREEMPT study demonstrated that the percent of patients with a higher than 50% reduction from baseline of the frequency of headache days was significantly greater for the OBOT-A-only group at week 56 [10]. A study analyzing long-term experience with OBOT-A in CM demonstrated that after an average of two years treatment with OBOT-A in responder CM patients the need for acute pain medication was decreased by 53% and emergency visits were reduced by 61% [77]. In a recent clinical trial, CM patients with medication overuse were studied after a five-day-long withdrawal period with 150 U OBOT-A injection, which confirmed the efficacy of OBOT-A [78]. Results of another clinical study in which OBOT-A was used in the dose of 155 U, in accordance with the PREEMPT study protocol, showed that the number of headache days per month and the acute medication intakes decreased significantly in the treatment group [79]. A recent prospective post-marketing cohort analysis in a real-life clinical setting demonstrated that OBOT-A significantly reduced the number of headache and migraine days, and showed a capability to increase the numbers of headache-free days [80].

### 3.6. Tolerability and Safety of OBOT-A in Treatment of CM

Based on the PREEMPT pooled data, the total treatment-related adverse events (AEs) of OBOT-A treatment in CM were 29.4% *vs.* 12.7% for placebo, and included neck pain (6.7% *vs.* 2.2%), muscular weakness (5.5% *vs.* 0.3%), eyelid ptosis (3.3% *vs.* 0.3%) and injection site pain (3.2% *vs.* 2.0%) [19]. These AEs were mild-to-moderate and transient. Serious AEs occurred in 4.8% of the CM patients on therapy compared to 2.3% for those in the placebo group, and no death was reported [19].

Data from pooled analysis of the safety and tolerability data of two phase 2 and two phase 3 double-blind, placebo-controlled trials (1997 CM patients, who received more than one dose of OBOT-A on average dose of 163 U per treatment cycle) revealed that the majority of the patients (72.9%) presented at least one AE. The most prevalent AEs were neck pain (12.6%), muscle weakness (8%), musculoskeletal stiffness (6.1%) and eyelid ptosis (4.6%) [81].

In view of immunogenicity, OBOT-A as a neurotoxin (bacterially derived protein) can be associated with the development of toxin-neutralizing antibodies. Based on the 496 analyzable plasma samples achieved from OBOT-A-treated patients, none was shown to be positive for neutralizing antibodies [8,19,81].

The above analysis confirmed the favorable safety and tolerability profile of OBOT-A in the prophylactic treatment of CM.

## 4. Discussion

CM is a highly disabling chronic pain syndrome, which represents the severe end of the episodic migraine spectrum. The chronification of migraine is influenced by medication overuse as well as psychiatric comorbidities, such as depression and anxiety. A cross-sectional study analyzed the comorbid psychiatric conditions, such as depression, stagnation syndrome and overattachment in chronic migraineurs [82]. The study confirmed that chronic migraineurs with higher perceived disability compared to those with lower perceived disability expressed higher depression scores on the Center For Epidemiologic Studies Depression Scale (21.61 + 8.98 *vs.* 10.63 + 4.79), as well higher stagnation scale scores (75.55 + 31.90 *vs.* 42.33 + 24.93) [82]. In a clinical study, women with CM with higher total scores on affective dysregulated temperamental component demonstrated higher scores on Beck Hopelessness Scale and Suicidal History Self-Rating Screening Scale [83]. However, the analysis of functional polymorphisms of genes (MAO-A3, CYP1A2*1F, GNB3) proposed to be associated with hopelessness and suicidal risk did not confirm associations [83]. Risk factors for chronification include young age at onset, frequent migraine attacks at baseline, mood disturbances (e.g., depression), as well as the presence of cutaneous allodynia [38]. To reduce the risk of migraine chronification, proper prophylactic anti-migraine drugs in appropriate doses should be used, and attention should also be paid to concomitant factors such as depression, hypertension and obesity [84]. When the episodic state of migraine is transformed to chronic state, treatment options become very limited. The observation originating from the cosmetic (anti-wrinkle) use of OBOT-A indicating that it may harbor therapeutic potential in headache disorders gave a new hope to CM sufferers. Several early reports opened up new fields of research concerning the potential modes of action of OBOT-A in the treatment of primary headaches.

The etiology of CM is supposed to be multifactorial with both genetic and environmental components proposed to play a role in it. The exact pathomechanism and genetic background of CM is still unknown; however, the overactivation of the TS, the central and peripheral sensitization processes as well as the effects of vasoactive neuropeptides may take a part in the pathogenesis. Based on the proposed pathomechanism, the inhibition of release of CGRP and other neuropeptides from the trigeminal system by OBOT-A provides a new possibility for the prophylactic treatment of CM. Recent clinical studies supposed that CGRP might be a potential biomarker of the responsiveness of CM patients to OBOT-A treatment [69]. The pooled analyses proved the favorable efficacy and good safety profile with low rate of AEs of OBOT-A therapy in CM. Furthermore, the results from clinical studies showed that OBOT-A was also effective and well tolerated in CM patients with comorbid depression [17]. Follow-up experience with OBOT-A suggests that three-quarters of CM patients have long-term response after one year [77]. The recommended injection schedule is every 12 weeks, however, the long-term follow-up revealed that after one year the injection regimen could be delayed, though not stopped [77]. Based on an Italian pilot study, CM has a huge economic impact. The study demonstrated that the direct mean annual cost of CM was 2250.0 euro *vs.* 523.6 euro per patient with episodic migraine [85]. Furthermore, a large study (the International Burden of Migraine Study) involving five European countries (UK, France, Germany, Italy and Spain) revealed that the total annual cost of the CM *vs.* episodic migraine was higher in all five countries [86]. A cost-effectiveness survey demonstrated that OBOT-A treatment reduced the headache days by 38 days per year that is associated with a high economic impact [87]. Important clinical aspects regarding the medical care of CM patients include not only the efficacy and safety of a drug, but also the responder and the discontinuation rates, as well as the health-related quality of life [88]. OBOT-A does fulfill the above criteria in the treatment of CM.

## 5. Conclusions

Up until today, there is no exact explanation for the pathomechanism of CM, and the establishment of an efficacious treatment for CM patients represents a big challenge for the both the researchers and the medical team. Overall, we can consider OBOT-A as an effective, safe and well-tolerable prophylactic treatment for CM. Currently, it is the one and only FDA-approved preventive medication for chronic migraineurs. The ultimate goals for the research in the field of primary headaches are to find their genetic backgrounds, to determine the full scope of their pathomechanism, and to expand the therapeutic options of OBOT-A in the different headache conditions.
